# *In-vitro* anticancer activity of *Rauvolfia tetraphylla* extract on mcf-7 breast cancer cell lines

**DOI:** 10.6026/97320630019043

**Published:** 2023-01-31

**Authors:** Abraham Sabu, Selvaraj J, Gayatridevi R, Dilipan E

**Affiliations:** 1 Department of Physiology, Saveetha Dental College and Hospitals, Saveetha Institute of Medical and Technical Sciences, Saveetha University, Chennai 600077, Tamil Nadu, India; 2 Department of Biochemistry, Saveetha Dental College and Hospitals, Saveetha Institute of Medical and Technical Sciences, Saveetha University, Chennai 600077, Tamil Nadu, India

**Keywords:** *Rauwolfia tetraphylla*, breast cancer, cytotoxicity, gene expression, TGF and Bcl1 gene

## Abstract

The medicinal herb *Rauwolfia tetraphylla* is utilized by South Indian tribes to treat various medical ailments, although its cytotoxicity action has not been studied. As a result, the emphasis of the current investigation is on the anticancer activity of *Rauvolfia tetraphylla* extracts on MCF-7 breast cancer cell lines, as well as their effects on the levels of gene expression for BCl2 and TGF. The study found that the anticancer activity of *R. tetraphylla* extract demonstrated significant cytotoxic activity against MCF-7 breast cancer cell lines. Because of this, its anticancer effect may be caused by apoptosis, which is caused by DNA breaking and is helped by active phytochemicals like alkaloids, phenols, and flavonoids in the extracts. It also promotes apoptosis by altering Bcl-2 and TGF expression levels. The present study suggests using *R. tetraphylla* extract as an anticancer agent in traditional medicine.

## Background:

Medicinal plants are vital to the worldwide survival of both human and animal populations. They are an abundant source of phytochemicals that may be utilized to treat various human illnesses [1, 2]. In India, cancer and other diseases have been treated for a long time with natural compounds made from medicinal plants [3]. However, the use of these medicinal plants as a medicine to treat different pathophysiological disorders requires rigorous validation and verification [4, 5,6]. There are an estimated 6,000 medicinal plants in India, of which only 3,000 have been shown to have medical potential, and many more remain unknown [7]. *Rauwolfia tetraphylla* L. (Family: Apocynaceae) is a tiny tree/shrub that grows to a maximum height of 6 feet. The tree has distinctive leaves that are generally medium to dark green in hue and whorled. Moreover, these trees are common in groups of four equal-sized plants per leaf node. These trees are also prevalent in India and are of importance owing to the distinctive indole alkaloids they contain [8]. The *R. tetraphylla* plant is commonly used by South-Indian tribes to treat many medical conditions. Many essential plant components, including roots, stems, bark, and leaves, are employed to extract and isolate the chemical elements [9]. *R. tetraphylla* has significant pharmacological action, making it useful in the treatment of a broad range of human health issues via antimicrobial [10], anti-inflammatory [9], anti-cancer [11], and antioxidant [12] activity. Breast cancer is the most complicated and heterogeneous condition affecting breast tissue. More than one million new instances of breast cancer are detected worldwide [13]. Available therapies for breast cancer include chemotherapy, radiation therapy, hormone therapy, targeted therapy, and surgery. The kind of therapy is determined by the type and stage of the cancer. However, cancer recurrence and different side effects are the greatest challenges connected with these medicines, and patients receiving treatment suffer from physical and psychological stress. BCL2 and TGF expression in breast cancer has been documented to be connected with a better prognosis in patients treated with hormones or chemotherapy, among several protein expression pathways in cancer [14, 15]. Even while these treatments are effective in inhibiting metastasis, they also have a considerable number of adverse effects since they harm normal cells and cancer cells [16]. These factors urge the quest for alternate, safer, and more efficient therapeutic procedures. Consequently, the current work examines the anticancer efficacy of *Rauvolfia tetraphylla* extracts on MCF-7 breast cancer cell lines and their impacts on BCl2 and TGF gene expression levels.

## Material and Methods:

## Chemicals:

We used materials acquired from Gibco (Canada) for this experiment, including trypsin-EDTA, foetal bovine serum (FBS), antibiotics/antimycotics, DMEM, and PBS. Both the JC-1 (5,5,6,6 - tetrachloro-1,1,3,3 -tetraethylbenzimidazolocarbocyanine iodide) and the real-time PCR kit (MESA Green) were obtained from Invitrogen, USA. Pure, analytical-grade chemicals were employed at every step of the process.

## Extract preparation:

The bioactive components of *Rauvolfia tetraphylla* were extracted with 70% ethanol using Soxhlet equipment. The extract was further filtered using Whatman No. 1 filter paper. The solvent was evaporated at low pressure using rotary evaporator equipment to get a viscous mass, which was then kept at 4°C until use.

## Procurement and culture of human breast cancer cell line MCF-7:

The MCF-7 cell line was obtained from the National Center for Cell Science (NCCS) in Pune, India, and grown according to the instructions supplied. MCF-7 cells were cultured in MEM with 10% FBS at 37 degrees Celsius in a 5% CO2 incubator.

## Cytotoxicity studies:

The human breast cancer cell line MCF-7 cell was seeded in 96-well plates at a density of 5x105 cells/well and allowed to adhere to the well overnight. After incubation, cultured cells were stimulated in triplicate with different doses of *R. tetraphylla* extracts and incubated for 24 hours at 37 °C in a 5% humidified CO2 incubator. Following that, 3-(4,5-dimethylthiazol2-yl)-2,5-diphenyltetrazolium bromide (MTT) was added to each well, and the incubation was extended for 4 hours at 37°C. The cells were resuspended in 200 µl dimethyl sulfoxide (DMSO) to dissolve the formazan produced by MTT. The optical density (OD) of the solution was measured using a spectrometer at 570 nm. The trials were carried out three times separately. Each replicate group's mean optical density (OD) ±SD was computed.

The inhibitory rate of cell growth was calculated using the equation:

% Growth inhibition = (1 - OD extract treated)/OD negative control x 100.

## Gene expression analysis by Real Time PCR:

Total RNA was extracted from treated cell cultures using Trizol reagent (Sigma). Total RNA (2 µg) from each sample was reverse transcribed according to the manufacturer's procedure using a commercial Superscript III first-strand cDNA synthesis kit (Invitrogen, USA). For the BCL2 and TGF genes, real-time PCR was performed on an MX3000p PCR machine (Stratagene, Europe). MESA Green PCR Master Mix was used for the reaction (it contains all the PCR components and SYBR green dye) Eurogentec, Inc., USA Melting curve analysis was used to assess the specificity of the amplification product for each primer combination. The data was processed using the comparative CT approach, and the fold change was determined using the 2CT method given by Schmittgen and Livak (2008) with CFX Manager Version 2.1 (Bio Rad, USA).

## Statistical analysis:

Data were presented as the means ±SD of three separate studies that were each carried out in triplicate. A one-way analysis of variance was used for the statistical analysis and a value of p<0.05 was used to indicate that the result was statistically significant.

## Results and Discussion:

**Figure 1:**
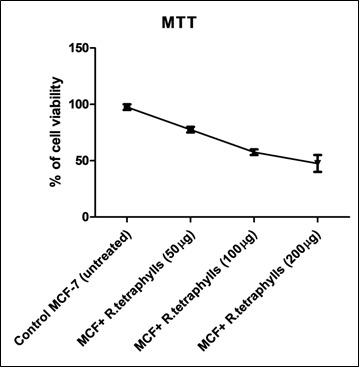
Dose dependant cytotoxicity effect of *R. tetraphylla* over cell viability.

**Figure 2:**
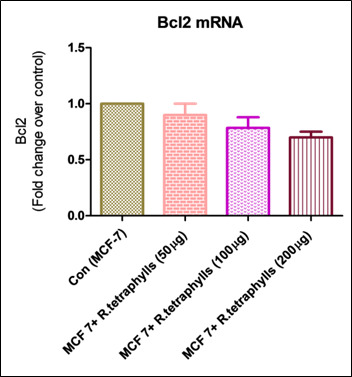
Effect of *R. tetraphylla* on Bcl-2 mRNA levels on MCF -7 cell lines. Each column represents mean ± SEM compared with control MCF-7 cell lines.

**Figure 3:**
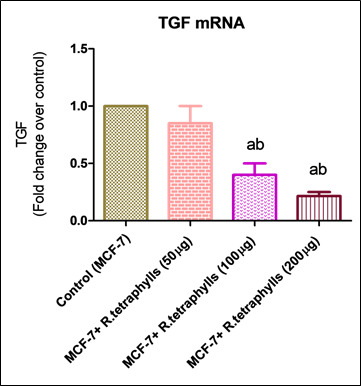
Effect of *R. tetraphylla* on TGF mRNA levels on MCF -7 cell lines. Each column represents mean ± SEM compared with control MCF-7 cell lines.

In this study, the plant *R. tetraphylla*, used in traditional medicine in different parts of South India to treat health problems, was looked at to find new drugs that might be better for treating cancer [17]. Even though five alkaloids extracted from *R. tetraphylla* were previously found to have no significant cytotoxicity against five human cancer cell lines, all with an IC50>40 µM [18], this plant has a large number of alkaloids. Because different parts of the plant might have cytotoxic properties, the leaves were separated using a bioassay and tested on cervical cancer cells and four other cancer cell lines. The MTT test was used to investigate the cytotoxic activity of R. tetraphylla extract on the MCF-7 cell line (Figure 1). At a dose of 100 µg/mL, the extract inhibited growth to a maximum of 57.5 ±3.5%. The plant extract was shown to be the most cytotoxic (at 100 µg/mL) in the MTT experiment. Although the American National Cancer Institute considers an extract to be a good candidate for future bioassay-guided analysis only if it has a significant cytotoxic effect with an IC50 of 30 µg/mL [19], we considered the possibility that the extract contains substances that interfere with the desired activity. Some active chemicals obtained from other species of Rauvolfia have been shown to produce cytotoxicity, lending credence to this theory [20, 21, 22]. Furthermore, reserpine (an indole alkaloid) extracted from Rauwolfia serpentina [22] and alstonine (a β-carboline alkaloid) isolated from Rauwolfia vomitoria [23] have cytotoxic action against prostate cancer cell lines (PC3 and LNCaP, respectively). This study aimed to determine the molecular mechanism by which apoptosis was triggered in MCF-7 cell lines by *R. tetraphylla* extract. The levels of expression of apoptosis-related genes, including Bcl-2 and TGF were analyzed, and it was shown that mRNA played a vital role in the process of apoptosis. The measurement of mRNA levels was carried out with the help of one step RT-PCR MESA green mix quantitative real time reverse transcription PCR. Compared with normal MCF-7 cell lines, the mRNA level of Bcl-2 in *R. tetraphylla* was shown to be considerably (p<0.01) up regulated. Compared with expression levels in normal MCF7 cell lines, the level of TGF produced by *R. tetraphylla* is considerably (p<0.001) lower (Figure 2, Figure 3).The most important proteins that control apoptosis in cancer are from the Bcl-2 family. These are used to treat different kinds of cancer and are an important part of chemotherapy for breast cancer. In addition, the Bcl-2 family participates in the control of apoptosis in various ways. These ways regulate the crucial last step of activating or inhibiting caspases, which is necessary for cell survival [24]. The likelihood of cells going through apoptosis is reduced when there is a mutation in the Bcl-2 gene [25, 26]. Apoptosis is caused when Bcl-2 expression levels drop below a certain threshold. The expression of Bcl-2 was discovered to be down regulated in cells that were treated with an extract of *R. tetraphylla*, which resulted in the cells having a slightly lower level. However, the expression of Bcl-2 was found to be upstream regulated in the MCF-7 cells that served as the control. TGF-is is also known to limit the growth of different cancer cell lines, one of which is the human breast cancer cell line MCF-7 [27]. The anticancer action of tamoxifen has been linked to the indirect stimulation of the TGF pathway and the induction of apoptosis via TGF [28]. There are a few different hypotheses floating around on how TGF works. Studies have shown a connection between its growth-inhibiting actions and the down-regulation of genes involved in cellular proliferation. These genes include those that encode cyclin-dependent kinases [29,30]. Based on the present work, it is tempting to believe that the downregulation of the TGF gene may play a significant part in the growth inhibition of the MCF-7 cell lines caused by the extract of *R. tetraphylla*.

## Conclusion:

The present study concluded that the anti-cancer activity of *R. tetraphylla* extract demonstrated significant cytotoxic activity against MCF-7 breast cancer cell lines. As a result, the mechanism of its anti-cancer effect may be apoptosis produced by DNA breakage, which is aided by active phytochemicals in the extracts, such as alkaloids, phenols, and flavonoids. It also promotes apoptosis by altering Bcl-2 and TGF expression levels. The present study warrants using *R. tetraphylla* extract as an anti-cancer agent in traditional medicine.
